# Bis(di­cyclo­hexyl­ammonium) sulfate dihydrate

**DOI:** 10.1107/S1600536814000968

**Published:** 2014-02-05

**Authors:** Daouda Ndoye, Mouhamadou M. Sow, Libasse Diop

**Affiliations:** a Laboratoire de Chimie Minerale et Analytique (LACHIMIA), Departement de Chimie, Faculte des Sciences et Techniques, Universite Cheikh Anta Diop, Dakar, Senegal

## Abstract

In the title dihydrate salt, 2C_12_H_24_N^+^·SO_4_^2−^·2H_2_O, the cation possesses twofold rotational symmetry, with the N atom situated on the twofold axis. The sulfate anion has fourfold roto-inversion symmetry, with the S atom located on the -4 axis. In the crystal, the components are linked *via* ammonium–sulfate N—H⋯O and water–sulfate O—H⋯O hydrogen bonds, forming a three-dimensional network.

## Related literature   

For the structure of tri­ammonium hydrogen di­sulfate, see: Suzuki & Makita (1978 ▶). For various sulfate complexes, see: Hathaway (1973 ▶); Diassé-Sarr *et al.* (1997 ▶); Diallo *et al.* (2010 ▶); Diop *et al.* (2012 ▶).
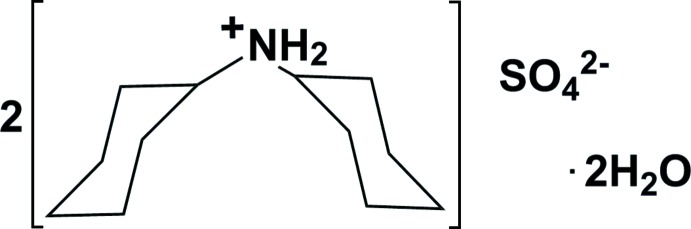


## Experimental   

### 

#### Crystal data  

2C_12_H_24_N^+^·SO_4_^2−^·2H_2_O
*M* *_r_* = 496.74
Tetragonal, 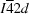

*a* = 12.437 (3) Å
*c* = 17.290 (4) Å
*V* = 2674.4 (11) Å^3^
*Z* = 4
Mo *K*α radiation
μ = 0.16 mm^−1^
*T* = 293 K
0.48 × 0.44 × 0.37 mm

#### Data collection  

Bruker APEXII CCD area-detector diffractometer
9860 measured reflections
1191 independent reflections
1131 reflections with *I* > 2σ(*I*)
*R*_int_ = 0.019

#### Refinement  

*R*[*F* ^2^ > 2σ(*F* ^2^)] = 0.026
*wR*(*F* ^2^) = 0.075
*S* = 1.16
1191 reflections
84 parameters
H atoms treated by a mixture of independent and constrained refinement
Δρ_max_ = 0.16 e Å^−3^
Δρ_min_ = −0.12 e Å^−3^
Absolute structure: Flack (1983 ▶); 514 Friedel pairs
Absolute structure parameter: 0.04 (10)

### 

Data collection: locally modified *CAD-4 Software* (Enraf–Nonius, 1989 ▶); cell refinement: *SET4* (de Boer & Duisenberg, 1984 ▶); data reduction: *HELENA* (Spek, 1997 ▶); program(s) used to solve structure: *SHELXS97* (Sheldrick, 2008 ▶); program(s) used to refine structure: *SHELXL97* (Sheldrick, 2008 ▶); molecular graphics: *PLATON* (Spek, 2009 ▶); software used to prepare material for publication: *SHELXL97* and *PLATON*.

## Supplementary Material

Crystal structure: contains datablock(s) I, New_Global_Publ_Block. DOI: 10.1107/S1600536814000968/pk2513sup1.cif

Structure factors: contains datablock(s) I. DOI: 10.1107/S1600536814000968/pk2513Isup2.hkl

Supporting information file. DOI: 10.1107/S1600536814000968/pk2513Isup3.cml

CCDC reference:

Additional supporting information:  crystallographic information; 3D view; checkCIF report

## Figures and Tables

**Table 1:** Hydrogen-bond geometry (Å, °)

*D*—H⋯*A*	*D*—H	H⋯*A*	*D*⋯*A*	*D*—H⋯*A*
N1—H1N⋯O1	0.929 (18)	1.919 (19)	2.8468 (17)	176.6 (16)
O1*W*—H1*W*⋯O1	0.93 (4)	2.12 (4)	3.020 (2)	163 (3)

**Fig. 1.**
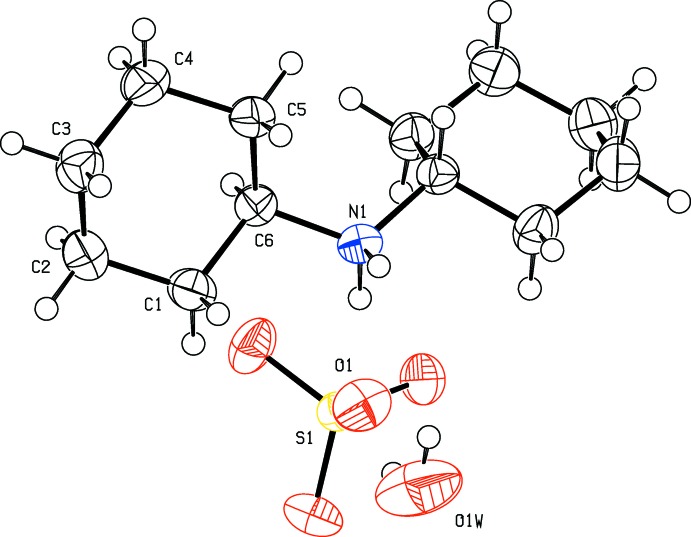
A view of the molecular structure of the title salt, with atom labelling. The displacement ellipsoids are drawn at the 50% probability level.

**Fig. 2.**
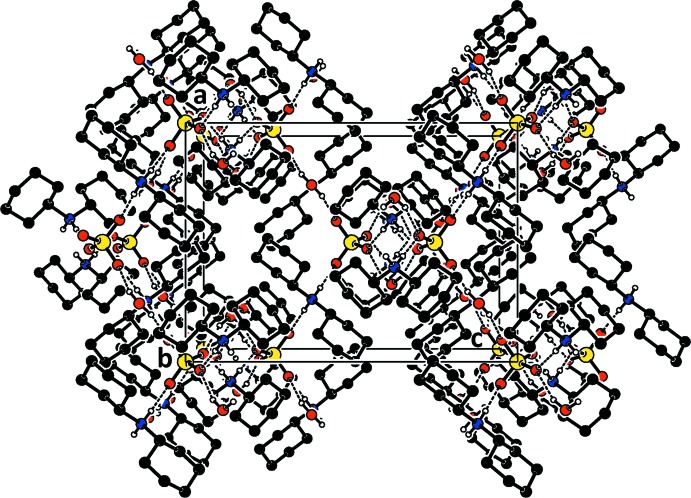
A view along the b axis of the crystal packing of the title salt. Hydrogen bonds are shown as dashed lines (see Table 1 for details).

## supplementary crystallographic information

### 1. Comment

A number of sulfato complexes have been synthesized and characterized in order to study the behaviour of the sulfate anion as a ligand (Hathaway, 1973). The triammonium hydrogen disulfate salt has been prepared by the reaction of ammonia with sulfuric acid (Suzuki & Makita, 1978). In our laboratory, previous work on the behaviour of the sulfate ion has been studied especially in relation to tin(IV) complexes (Diassé-Sarr *et al.*, 1997; Diallo *et al.*, 2010; Diop *et al.*, 2012). In the present work, we prepared the title salt by the reaction of aminoiminomethanesulfonic acid and dicyclohexylamine, and we describe herein its crystal structure.

The molecular structure of the title salt is illustrated in Fig. 1. The dicyclohexylammonium cation possesses two-fold rotational symmetry, with atom N1 situated on the two-fold axis. The sulfate cation has fourfold rotary inversion symmetry with atom S1 located on the 4.

In the crystal, the various units are linked *via* N—H···O(sulfate) and O—H(water)···O(sulfate) hydrogen bonds forming a three-dimensional network (Table 1 and Fig. 2).

### 2. Experimental

The title compound was obtained by reacting aminoiminomethanesulfonic acid with dicyclohexylamine in a 1:1 molar ratio in water. The solution was heated for 2 h, stirred for *ca* 8 h and then filtered. The filtrate was allowed to evaporation in a drying cupboard at 333 K, and yielded colourless block-like crystals of the title salt suitable for an X-ray diffraction analysis.

### 3. Refinement

The NH_2_ and water H atoms were located in a difference Fourier map. The NH_2_ H atom (the N atom is located on a two-fold axis) was freely refined while the water H atom (the O atom is located on the two-fold axis) was refined with *U*_iso_(H) = 1.5*U*_eq_(O). The C-bound H atoms were included in calculated positions and treated as riding atoms: C—H = 0.95 Å with *U*_iso_(H) = 1.2*U*_eq_(C).

### Crystal data

**Table:**

2C_12_H_24_N^+^·SO_4_^2^^−^·2H_2_O	*D*_x_ = 1.234 Mg m^−^^3^
*M**_r_* = 496.74	Mo *K*α radiation, λ = 0.71073 Å
Tetragonal, *I*42*d*	Cell parameters from 5803 reflections
Hall symbol: I -4 2bw	θ = 3.3–25.0°
*a* = 12.437 (3) Å	µ = 0.16 mm^−^^1^
*c* = 17.290 (4) Å	*T* = 293 K
*V* = 2674.4 (11) Å^3^	Prism, colourless
*Z* = 4	0.48 × 0.44 × 0.37 mm
*F*(000) = 1096	

### Data collection

**Table:**

Bruker APEXII CCD area-detector diffractometer	1131 reflections with *I* > 2σ(*I*)
Radiation source: Rotating Anode	*R*_int_ = 0.019
Graphite monochromator	θ_max_ = 25.0°, θ_min_ = 2.0°
ω scans	*h* = −14→14
9860 measured reflections	*k* = −14→14
1191 independent reflections	*l* = −20→20

### Refinement

**Table:**

Refinement on *F*^2^	Secondary atom site location: difference Fourier map
Least-squares matrix: full	Hydrogen site location: inferred from neighbouring sites
*R*[*F*^2^ > 2σ(*F*^2^)] = 0.026	H atoms treated by a mixture of independent and constrained refinement
*wR*(*F*^2^) = 0.075	*w* = 1/[σ^2^(*F*_o_^2^) + (0.0427*P*)^2^ + 0.6142*P*] where *P* = (*F*_o_^2^ + 2*F*_c_^2^)/3
*S* = 1.16	(Δ/σ)_max_ = 0.001
1191 reflections	Δρ_max_ = 0.16 e Å^−^^3^
84 parameters	Δρ_min_ = −0.12 e Å^−^^3^
0 restraints	Absolute structure: Flack (1983); 514 Friedel pairs
Primary atom site location: structure-invariant direct methods	Absolute structure parameter: 0.04 (10)

### Special details

**Table:**

Geometry. Bond distances, angles *etc*. have been calculated using the rounded fractional coordinates. All su's are estimated from the variances of the (full) variance-covariance matrix. The cell e.s.d.'s are taken into account in the estimation of distances, angles and torsion angles
Refinement. Refinement of *F*^2^ against ALL reflections. The weighted *R*-factor *wR* and goodness of fit *S* are based on *F*^2^, conventional *R*-factors *R* are based on *F*, with *F* set to zero for negative *F*^2^. The threshold expression of *F*^2^ > σ(*F*^2^) is used only for calculating *R*-factors(gt) *etc*. and is not relevant to the choice of reflections for refinement. *R*-factors based on *F*^2^ are statistically about twice as large as those based on *F*, and *R*- factors based on ALL data will be even larger.

### Fractional atomic coordinates and isotropic or equivalent isotropic displacement parameters (Å^2^)

**Table:**

	*x*	*y*	*z*	*U*_iso_*/*U*_eq_	
N1	0.10093 (14)	0.25000	0.12500	0.0327 (5)	
C1	0.08284 (13)	0.36633 (14)	0.01210 (9)	0.0436 (5)	
C2	0.14068 (15)	0.43411 (15)	−0.04852 (10)	0.0503 (6)	
C3	0.21449 (14)	0.51624 (14)	−0.01157 (10)	0.0492 (5)	
C4	0.29313 (14)	0.46346 (14)	0.04327 (11)	0.0493 (5)	
C5	0.23596 (13)	0.39552 (13)	0.10417 (9)	0.0414 (5)	
C6	0.16348 (12)	0.31304 (12)	0.06577 (8)	0.0330 (4)	
S1	0.00000	0.00000	0.00000	0.0307 (1)	
O1	−0.02822 (9)	0.09357 (10)	0.04872 (7)	0.0507 (4)	
O1W	−0.1807 (2)	0.25000	0.12500	0.1049 (13)	
H1A	0.03480	0.41170	0.04200	0.0520*	
H1N	0.0568 (15)	0.2011 (15)	0.0997 (10)	0.045 (5)*	
H2A	0.18250	0.38740	−0.08190	0.0600*	
H2B	0.08790	0.47090	−0.08030	0.0600*	
H3A	0.25390	0.55400	−0.05160	0.0590*	
H3B	0.17180	0.56860	0.01650	0.0590*	
H4A	0.33540	0.51850	0.06880	0.0590*	
H4B	0.34190	0.41820	0.01400	0.0590*	
H5A	0.28880	0.35910	0.13600	0.0500*	
H5B	0.19330	0.44170	0.13740	0.0500*	
H6	0.20800	0.26330	0.03570	0.0400*	
H21B	0.03990	0.31170	−0.01340	0.0520*	
H1W	−0.143 (3)	0.203 (3)	0.093 (2)	0.1570*	

### Atomic displacement parameters (Å^2^)

**Table:**

	*U*^11^	*U*^22^	*U*^33^	*U*^12^	*U*^13^	*U*^23^
N1	0.0290 (9)	0.0315 (9)	0.0377 (9)	0.0000	0.0000	−0.0034 (8)
C1	0.0397 (8)	0.0456 (9)	0.0454 (9)	−0.0032 (7)	−0.0098 (7)	0.0033 (7)
C2	0.0534 (11)	0.0556 (11)	0.0419 (8)	−0.0025 (8)	−0.0062 (8)	0.0093 (8)
C3	0.0541 (10)	0.0414 (9)	0.0520 (9)	−0.0032 (7)	0.0021 (8)	0.0081 (8)
C4	0.0415 (9)	0.0496 (10)	0.0567 (9)	−0.0107 (7)	−0.0021 (8)	0.0092 (8)
C5	0.0369 (8)	0.0453 (9)	0.0419 (8)	−0.0076 (7)	−0.0062 (7)	0.0042 (7)
C6	0.0322 (7)	0.0320 (7)	0.0348 (7)	0.0015 (6)	0.0011 (6)	−0.0006 (6)
S1	0.0294 (2)	0.0294 (2)	0.0332 (3)	0.0000	0.0000	0.0000
O1	0.0526 (8)	0.0448 (7)	0.0546 (6)	−0.0044 (5)	0.0049 (5)	−0.0205 (6)
O1W	0.0498 (14)	0.109 (2)	0.156 (3)	0.0000	0.0000	−0.003 (2)

### Geometric parameters (Å, º)

**Table:**

S1—O1^i^	1.4789 (13)	C4—C5	1.526 (2)
S1—O1^ii^	1.4789 (13)	C5—C6	1.518 (2)
S1—O1^iii^	1.4789 (13)	C1—H21B	0.9700
S1—O1	1.4789 (13)	C1—H1A	0.9700
O1W—H1W^iv^	0.93 (4)	C2—H2B	0.9700
O1W—H1W	0.93 (4)	C2—H2A	0.9700
N1—C6^iv^	1.5062 (17)	C3—H3A	0.9700
N1—C6	1.5062 (17)	C3—H3B	0.9700
N1—H1N	0.929 (18)	C4—H4A	0.9700
N1—H1N^iv^	0.929 (18)	C4—H4B	0.9700
C1—C6	1.519 (2)	C5—H5B	0.9700
C1—C2	1.525 (2)	C5—H5A	0.9700
C2—C3	1.515 (3)	C6—H6	0.9800
C3—C4	1.512 (3)		
			
O1^i^—S1—O1^iii^	110.56 (7)	C6—C1—H1A	110.00
O1^ii^—S1—O1^iii^	108.93 (6)	C1—C2—H2A	109.00
O1—S1—O1^ii^	110.56 (7)	C1—C2—H2B	109.00
O1—S1—O1^iii^	108.93 (6)	C3—C2—H2A	109.00
O1—S1—O1^i^	108.93 (6)	C3—C2—H2B	109.00
O1^i^—S1—O1^ii^	108.93 (6)	H2A—C2—H2B	108.00
H1W—O1W—H1W^iv^	120 (3)	C2—C3—H3A	109.00
C6—N1—C6^iv^	117.81 (14)	C2—C3—H3B	109.00
C6—N1—H1N^iv^	106.5 (11)	C4—C3—H3A	109.00
C6—N1—H1N	109.0 (11)	C4—C3—H3B	109.00
C6^iv^—N1—H1N	106.5 (11)	H3A—C3—H3B	108.00
H1N—N1—H1N^iv^	107.6 (16)	H4A—C4—H4B	108.00
C6^iv^—N1—H1N^iv^	109.0 (11)	C3—C4—H4A	109.00
C2—C1—C6	110.46 (13)	C3—C4—H4B	109.00
C1—C2—C3	111.64 (14)	C5—C4—H4A	109.00
C2—C3—C4	111.33 (15)	C5—C4—H4B	109.00
C3—C4—C5	111.82 (14)	C4—C5—H5A	110.00
C4—C5—C6	110.43 (13)	C4—C5—H5B	110.00
N1—C6—C5	111.16 (11)	C6—C5—H5A	110.00
C1—C6—C5	111.37 (13)	C6—C5—H5B	110.00
N1—C6—C1	107.55 (12)	H5A—C5—H5B	108.00
H1A—C1—H21B	108.00	N1—C6—H6	109.00
C6—C1—H21B	110.00	C1—C6—H6	109.00
C2—C1—H1A	110.00	C5—C6—H6	109.00
C2—C1—H21B	110.00		
			
C6^iv^—N1—C6—C1	178.50 (10)	C1—C2—C3—C4	54.74 (19)
C6^iv^—N1—C6—C5	−59.34 (14)	C2—C3—C4—C5	−54.68 (19)
C6—C1—C2—C3	−55.56 (19)	C3—C4—C5—C6	55.35 (18)
C2—C1—C6—N1	178.63 (12)	C4—C5—C6—N1	−176.33 (12)
C2—C1—C6—C5	56.60 (17)	C4—C5—C6—C1	−56.41 (17)

Symmetry codes: (i) *y*, −*x*, −*z*; (ii) −*x*, −*y*, *z*; (iii) −*y*, *x*, −*z*; (iv) *x*, −*y*+1/2, −*z*+1/4.

### Hydrogen-bond geometry (Å, º)

**Table:**

*D*—H···*A*	*D*—H	H···*A*	*D*···*A*	*D*—H···*A*
N1—H1*N*···O1	0.929 (18)	1.919 (19)	2.8468 (17)	176.6 (16)
O1*W*—H1*W*···O1	0.93 (4)	2.12 (4)	3.020 (2)	163 (3)
